# Developing a xylanase XYNZG from *Plectosphaerella cucumerina* for baking by heterologously expressed in *Kluyveromyces lactis*

**DOI:** 10.1186/s12896-014-0107-7

**Published:** 2014-12-16

**Authors:** Fei Xiang Zhan, Qin Hong Wang, Si Jing Jiang, Yu Ling Zhou, Gui Min Zhang, Yan He Ma

**Affiliations:** Hubei Collaborative Innovation Center for Green Transformation of Bio-Resources, College of Life Science, Hubei University, Wuhan, 430062 People’s Republic of China; Tianjin Institutes of Industrial Biotechnology, Chinese Academy of Science, Tianjin, 300308 China

**Keywords:** Xylanase, Heterologous expression, *Kluyveromyces lactis*, Baking

## Abstract

**Background:**

Xylanase can replace chemical additives to improve the volume and sensory properties of bread in the baking. Suitable baking xylanase with improved yield will promote the application of xylanase in baking industry. The xylanase XYNZG from the *Plectosphaerella cucumerina* has been previously characterized by heterologous expression in *Pichia pastoris*. However, *P. pastoris* is not a suitable host for xylanase to be used in the baking process since *P. pastoris* does not have GRAS (Generally Regarded As Safe) status and requires large methanol supplement during the fermentation in most conditions, which is not allowed to be used in the food industry. *Kluyveromyces lactis*, as another yeast expression host, has a GRAS status, which has been successfully used in food and feed applications. No previous work has been reported concerning the heterologous expression of xylanase gene *xynZG* in *K. lactis* with an aim for application in baking.

**Results:**

The xylanase gene *xynZG* from the *P. cucumerina* was heterologously expressed in *K. lactis*. The recombinant protein XYNZG in *K. lactis* presented an approximately 19 kDa band on SDS-PAGE and zymograms analysis. Transformant with the highest halo on the plate containing the RBB-xylan (Remazol Brilliant Blue-xylan) was selected for the flask fermentation in different media. The results indicated that the highest activity of 115 U/ml at 72 h was obtained with the YLPU medium. The mass spectrometry analysis suggested that the hydrolytic products of xylan by XYNZG were mainly xylobiose and xylotriose. The results of baking trials indicated that the addition of XYNZG could reduce the kneading time of dough, increase the volume of bread, improve the texture, and have more positive effects on the sensory properties of bread.

**Conclusions:**

Xylanase XYNZG is successfully expressed in *K. lactis*, which exhibits the highest activity among the published reports of the xylanase expression in *K. lactis*. The recombinant XYNZG can be used to improve the volume and sensory properties of bread. Therefore, the expression yield of recombinant XYNZG can be further improved through engineered strain containing high copy numbers of the XYNZG, and optimized fermentation condition, making bread-baking application possible.

## Background

Nowadays, more and more attentions are paid on food safety and nutrition, and providing non-contaminated and fiber-rich food is becoming an important public issue. Accordingly, developing improved and new methods to produce whole wheat bread and reduce the use of chemical additives are challenges for the baking industry [1]. Traditional chemical food additives have been used in the baking industry to enlarge loaf volume, lengthen shelf life, and improve the taste of breads, etc. However, some of these compounds may threaten the health of consumers. For example, potassium bromate, the most widely used food additive, is now known to be a human carcinogen and has been banned by most of countries [2]. Azodicarbonamide, a bleaching and improving agent, is only a permitted food additive in certain countries. It partially degrades under the heat process to form trace amounts of semicarbazide, which shows carcinogenicity and has been proved to cause tumors [1]. Therefore, it is very urgent to find safe food additives to replace the previous harmful chemical additives. Recombinant enzymes, as safe substituents, were firstly applied in baking industry in the 1970s due to the ever-increasing demand for more natural products. In the past 40 years, many enzymes, including α-amylase, cellulase, hemicellulase, and xylanase, have been successfully applied in the baking industry [2,3].

Xylanase is used as baking additives to improve processing and product quality. It affects enhancements in dough and bread quality leading to improved dough flexibility, machinability and stability as well as a larger loaf volume and an improved crumb structure. However, xylanase has not been applied extensively in baking industry because of high cost and poor effects, especially in the developing country. To reduce the price of enzymes, it is highly desirable to adopt gene engineering to produce enzymes with better performance. Thus, hundreds of xylanase genes from bacteria, fungi and actinomycetes have been cloned and expressed heterolougously [4,5].

Now, many xylanases have been used to efficiently express in the yeast host, especially in *Pichia pastoris* within the past teens years. However, *P. pastoris* cannot be used in the food industry since it does not have GRAS status by the FDA (US Food and Drug Administration) and requires large methanol supplement during the fermentation in most conditions. Contrarily, *Kluyveromyces lactis*, as another yeast expression host, has a GRAS status by FDA, which permits its use in food and feed applications. Meanwhile, compared with other yeast expression systems, *K. lactis* has advantages of multicopy gene integration, easy genetic manipulation, the availability of a fully sequenced genome [6], and it can easily grow to a high density on inexpensive lactose-based media [7]. Therefore, *K. lactis* has been used to efficiently express many proteins in the past few years, and the best example for its use is commercial production of the milk clotting enzyme, bovine chymosin [7]. Thus, several researchers tried to express different xylanase in this system. XynAs from the extreme thermophile *Thermotoga* sp. strain FjSS3B.1 [8], and the *Dictyoglomus thermophilum* strain Rt46B.1 [9], respectively, and Xyn11A from *Bacillus halodurans* strain C-125 [10] were expressed in *K. lactis* using episomal vector. However, they would have the risk of instability due to lacking of selective pressure. Only XynB from the *T. maritima* MSB8 was expressed stable in *K. lactis* based on the integration vector [11]. However, these xylanases were not investigated about application in baking industry. Additionally, thermophilic and halophilic xylanases are not favorable in the dough baking since high temperature and salt are not necessary.

Xylanase gene *xynZG* was cloned from *P. cucumerina* and had been successfully expressed in *P. pastoris* in our previous report [12]. Recombinant xylanase XYNZG has high specific activity and the optimal pH and temperature of 6 and 40°C, respectively [12]. These features may indicate that XYNZG has the potential use in baking. Thus, we try to express xylanase gene *xynZG* in *K. lactis* and study the effect of recombinant XYNZG on dough and bread in bread-making. Additionally, xylo-oligosaccharides (XOs) of the hydrolytic products were recently reported to possess a remarkable potential for stimulating the growth of intestinal bifidobacteria and promoting the intestinal health [13]. Because of this reason, we investigated the hydrolytic products of XYNZG in this study.

## Results

### Construction of plasmid pKLAC2-*xynZG* and expression in *K. lactis*

**Figure 1:**
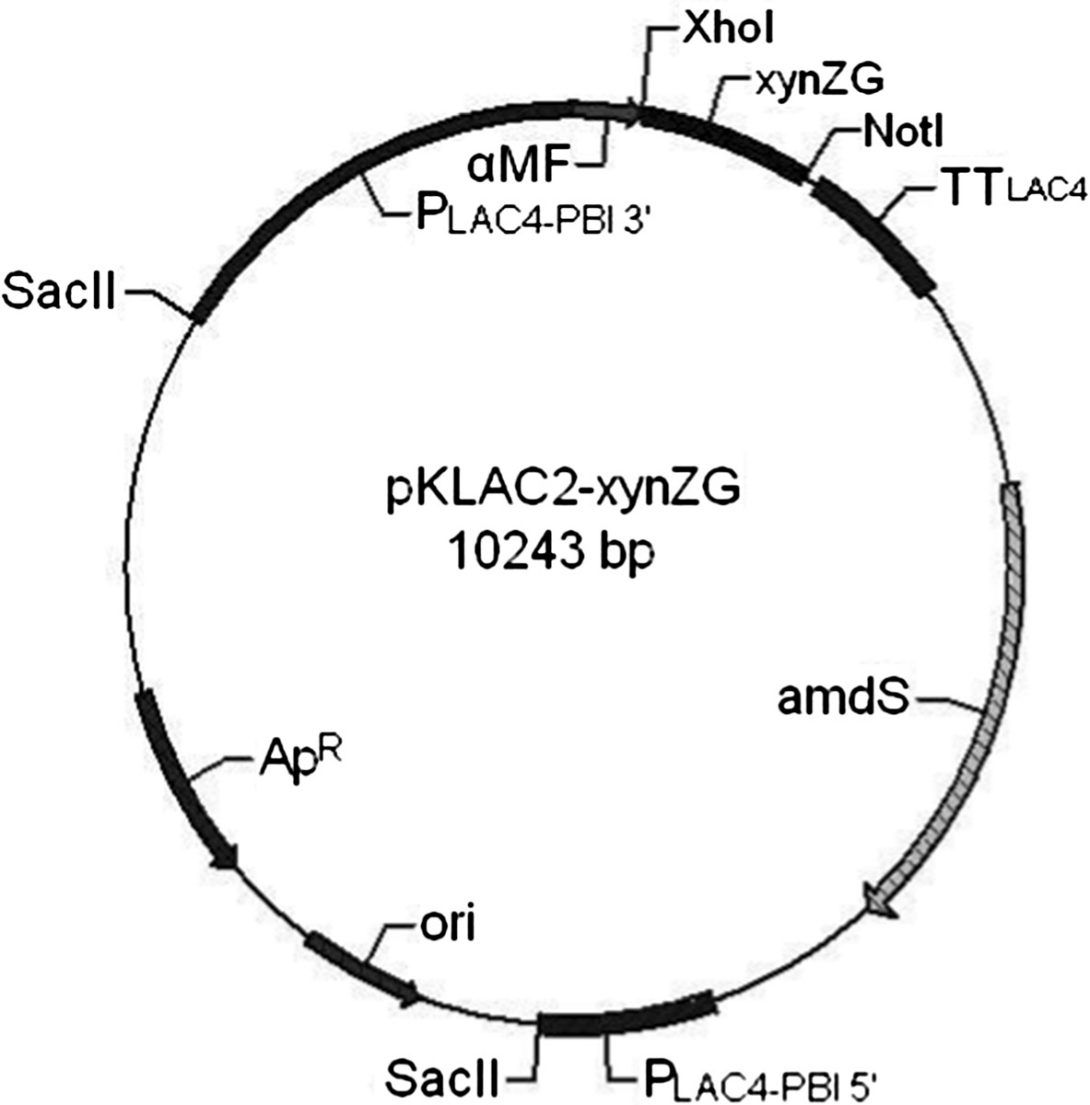
**The map for the expression plasmid pKLAC2-** ***xynZG*** **in** ***K. lactis*** **.** xynZG (the target gene encoding XYNZG), P_LAC4-PBI_ (LAC4 Promoter), α-MF (α-mating factor secretion leader sequence), Ap^R^ (Ampicillin resistance gene), amdS (encoding acetamidase to break down acetamide for use as a nitrogen source), ori (origin of *E. coli*), TT_LAC4_ (LAC4 transcription terminator).

**Figure 2:**
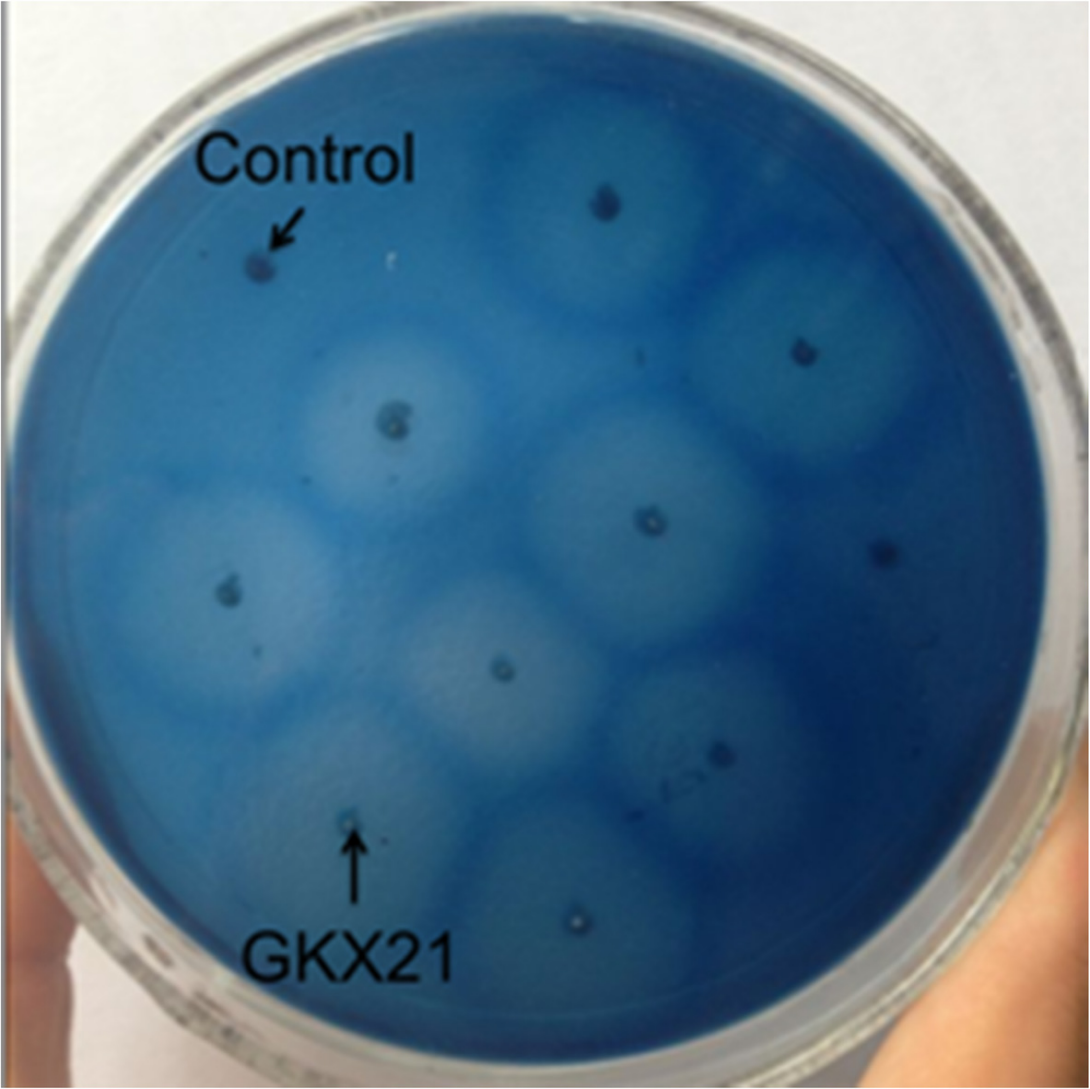
**The transformants of activity screening.** The transformants were cultured on the YPD plates containing 1% RBB xylan. The plates were photographed after 12 h incubation at 28°C. Negative control is *K. lactis* transformant harboring pKLAC2.

**Figure 3:**
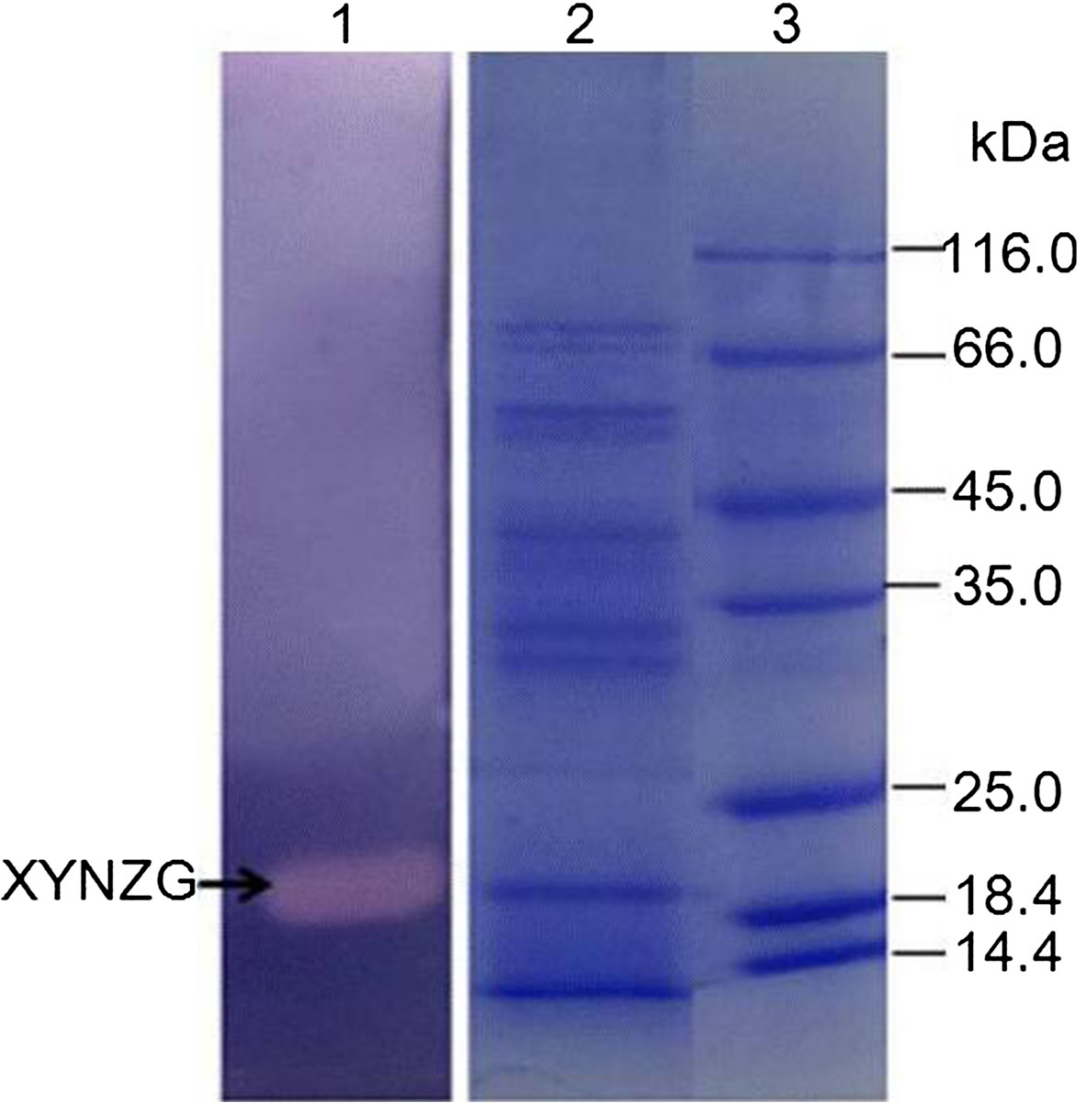
**SDS-PAGE and zymogram analysis of XYNZG.** Lane 1, zymogram analysis of the fermentation supernatant. Lane 2, the fermentation supernatant. Lane 3, the protein marker.

A 600 bp DNA fragment corresponding to *xynZG* was cloned into the pKLAC2 vector, fusing with the MF-alpha leader sequence for secretory expression in *K. lactis* GG799. The recombinant plasmid was named as pKLAC2-*xynZG* (Figure 1). For bio-safety and integration consideration, the ampicillin resistance gene on recombinant plasmid pKLAC2-*xynZG* was removed by *Sac*II digestion before transformation, so that the engineering strain producing xylanase does not contain any known antibiotic resistance genes. This is of vital importance for food enzymes [14]. The linearized plasmid was transformed into *K. lactis* and the transformants were cultured on the YPD plates containing 1% RBB xylan (Figure 2). One transformant with the largest halo GKX21 was chosen for shake-flask fermentation. The supernatant of the fermentation was performed on a 12% SDS-PAGE gel and the resolved proteins were visualized by staining with Coomassie brilliant blue. A band of proximately 19 kDa was observed, which accorded with the deduced molecular weight of XYNZG from *P. pastoris*. In addition, to detect the activity in situ and identify the target protein, the zymogram analysis was performed and the result revealed that there was only one about 19 kDa active band detected by Amido Black staining (Figure 3), consistent with our previous report [12].

### The fermentation of XYNZG in different media

**Figure 4:**
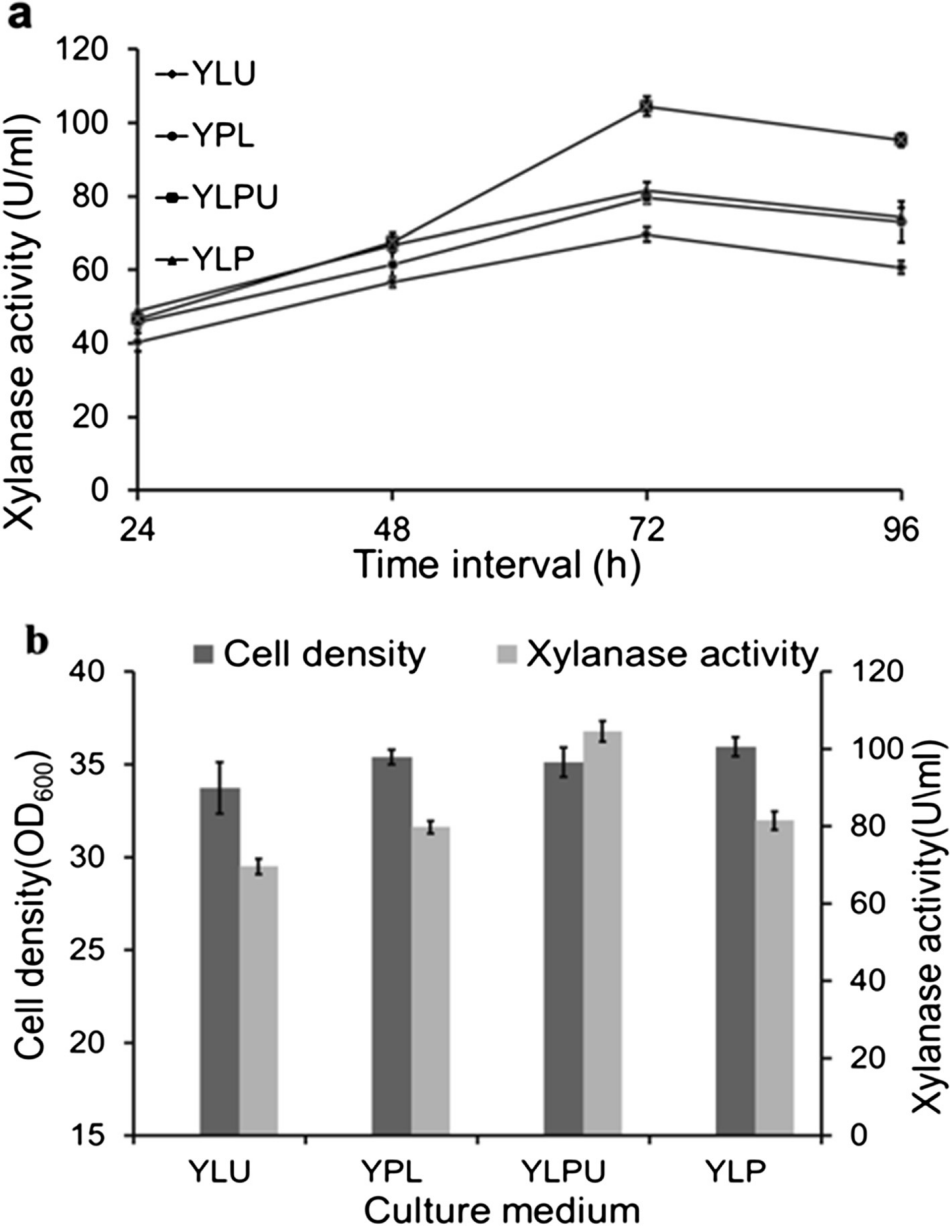
**The fermentation of xylanase XYNZG in different media. a**: The activity changes of XYNZG in YPL, YLP, YLU and YLPU medium during 96 h fermentation. The samples were taken and measured every 24 h. **b**: The comparison of biomass and activities at 72 h culture. The experiments were performed in triplicate.

The recombinants GKX21 were inoculated into YPL, YLP, YLU and YLPU medium, respectively, and underwent further shaken flask fermentation for 96 h. The activities and biomass were all measured during 96 h (Figure 4a) and the results indicated the activity of XYNZG reached highest level at 72 h with 115 U/ml in YLPU. In order to illustrate the relationship between the activities and the biomass, Figure 4b showed the biomass and enzyme activities in different media. No obvious biomass difference existed in the four media whereas the enzyme activity differed significantly. Thus, the medium YLPU was the optimal medium for *xynZG* expression in *K. lactis*.

### Mass spectrometry analysis of xylan hydrolysate

**Figure 5:**
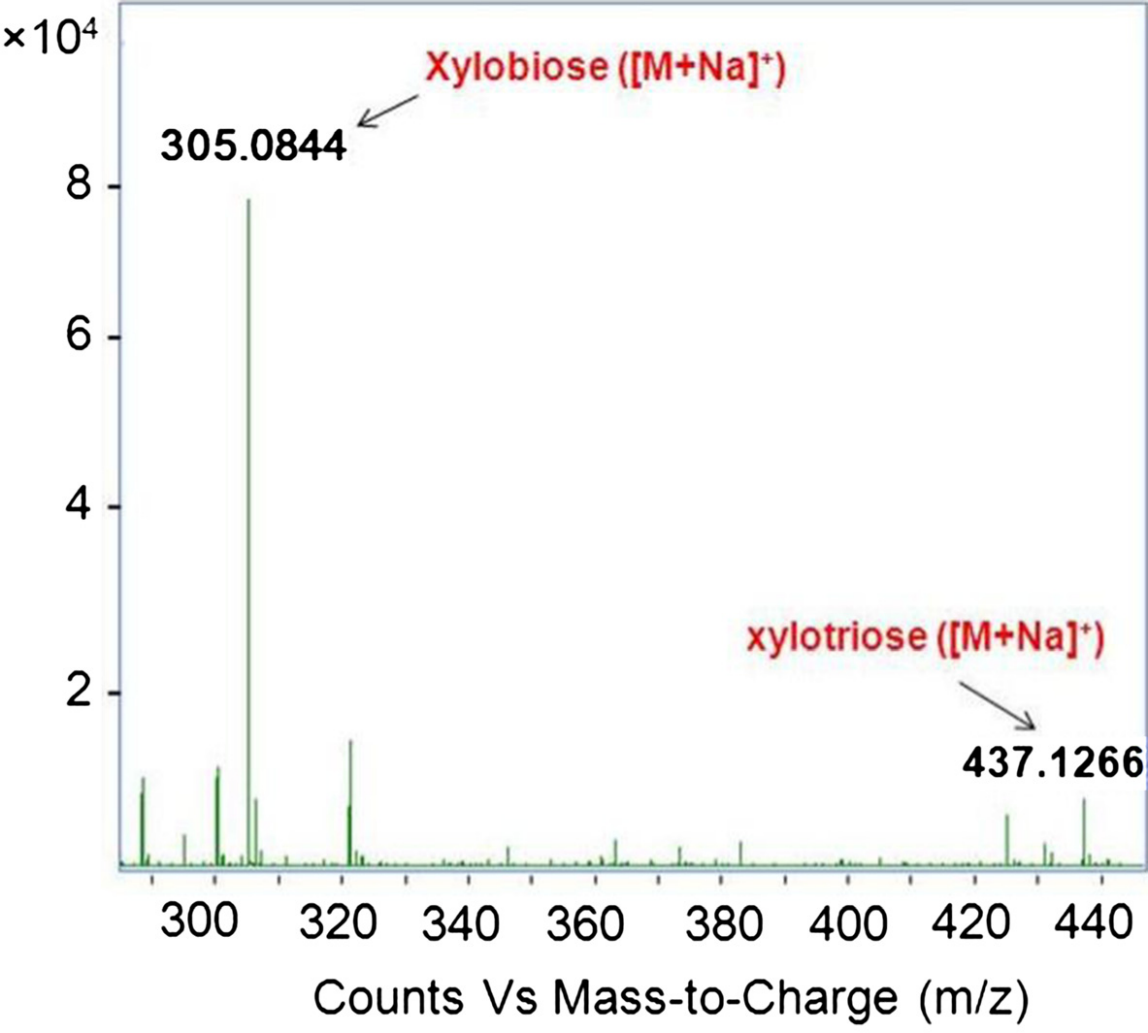
**Mass spectrometry analysis of xylan hydrolysate.**

The hydrolyzate was analyzed using electrospray ion source mass spectrometry in the positive ion reflective mode. As shown in Figure 5, there were two *m/z* peaks of 305.08, and 437.12, which corresponded to xylobiose ([M+Na]^+^) and xylotriose ([M+Na]^+^), respectively. Therefore, the results showed that xylobiose and xylotriose were the main hydrolysis products released from beechwood xylan by XYNZG and the yield of xylobiose was greater than that of xylotriose.

### Effects of XYNZG on the bread making

XYNZG has a broad working temperature range, which has an apparent optimal temperature of 40°C and retains approximately 75% and 55% of its maximum activity at 35°C and 25°C, respectively [12]. The mesophilic property of XYNZG makes it exhibit different characteristics from other fungi family11 xylanase, thus it may be highly suited for use in the baking industry as it is generally optimally active at the room temperatures most frequently used for dough preparation and proofing [2].

Bread quality assessment by sensory properties is largely based on personal judgment and subjective qualitative evaluation, whereas the results cannot be absolute but reflect the influences of consumer preferences [15]. The effects of XYNZG at different concentration on the attributes of dough or bread were shown in Table 1. Compared to the control lacking of xylanase, the dough handling time reduced from 6.5 min to 6.0 min and the height-diameter ratio of dough increased with the increasing concentration of XYNZG. Meanwhile, the addition of xylanase remarkably modified organoleptic properties of fresh whole wheat bread, so that the total sensory properties score reached 93 when 1200 ppm of 50 U XYNZG was supplemented, while the control only got a score of 85. However, the dough handling properties became a little sticky when the concentration of XYNZG reached 2000 ppm which was not suitable for manufacturing conditions which required the dough to be more 'machine-friendly' to avoid sticking to machinery parts. The result was consistent with the previous report that extra addition of the xylanase was not necessary in the baking process [16]. Thus, the proper concentration of XYNZG was 1200 ppm.

**Table 1:** **Effects of xylanase XYNZG on dough and bread properties**

**Trial number**	**Maximal score**	**1**	**2**	**3**	**4**
50 U xylanase (ppm)		0	800	1200	2000
Kneading dough time (min)		6.5	6.0	6.0	6.0
Height-diameter ratio of round bread		6.28/10.7	6.40/11.0	6.46/11	6.33/11.52
Dough handling properties		Inadhesion	Inadhesion	Inadhesion	A little stickiness
Volume of hand bag	35	25	30	32	33
Color of crust	10	8	8	8	8
Color of crumb	10	8	8	8	8
Skin and shape	5	3	4	4	4
Smoothness	10	8	8	8	8
Texture structure	25	22	21	20	19
Springiness	10	7	9	9	9
Taste	5	4	4	4	4
Total score	100	85	92	93	93

The experiment was performed by one step fermentation. Water, yeast, sodium chloride, modifier and oil bread were mixed according to the fixed ratio, except for XYNZG. The dough was proofed at 38°C for 150 min, and baked at 225°C for 20 min. Static (texture profile analysis, firmness and relaxation test), image analysis, sensory analysis and color measurements (colorimeter and photoshop system) were used for bread quality evaluation.

**Figure 6:**
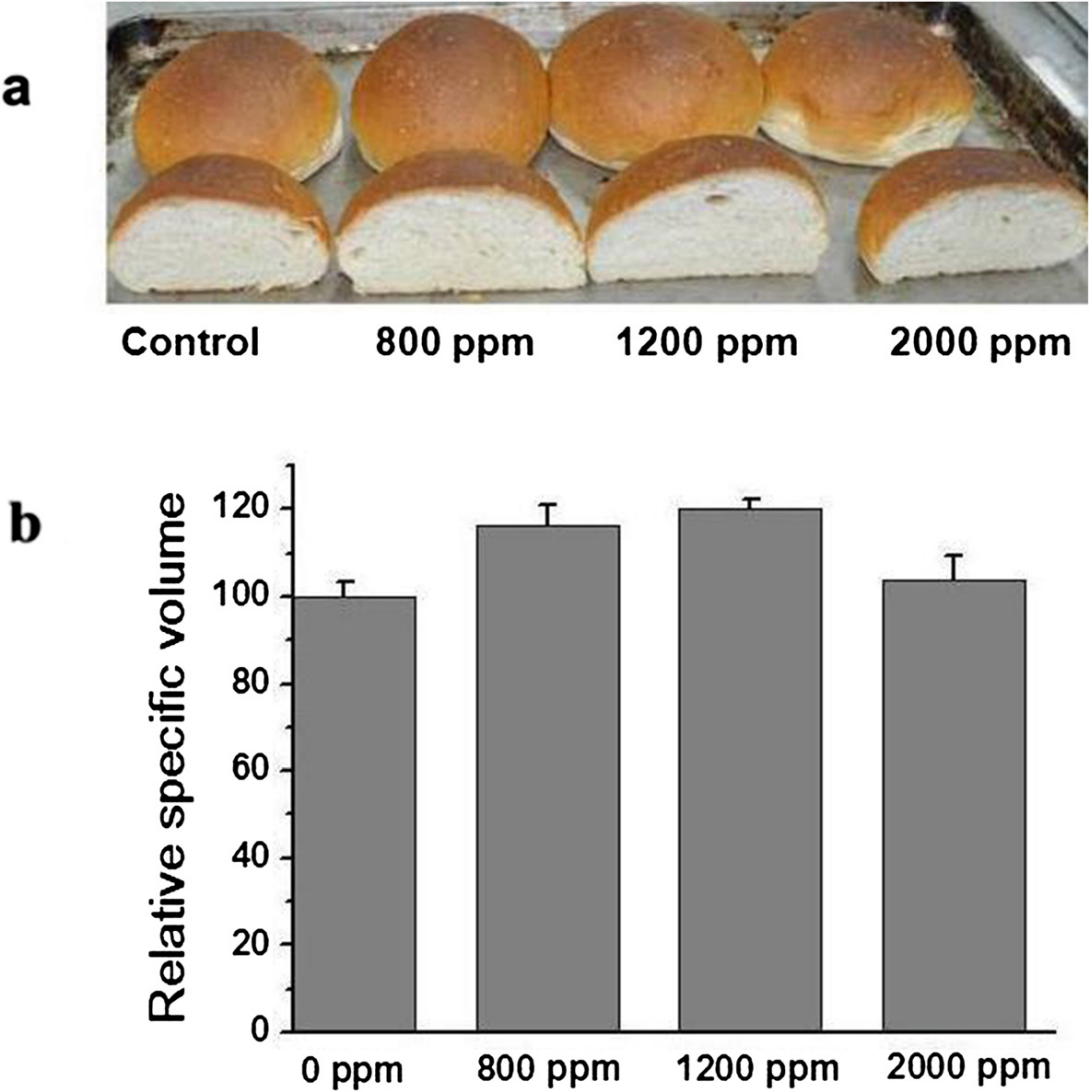
**Pictorial showing the effects of various concentrations of XYNZG on the volume of wheat bread. a**: Photograph of bread and cut bread. **b**: The relative specific volume of bread. Data represent mean±standard deviation of three replicates. The activity of XYNZG was 50 U/ml.

In order to avoid the personal sensory difference, the volume and texture of bread were also evaluated by the Volume Measuring Device and the Texture Analyzer, respectively. As shown in Figure 6, the addition of XYNZG to the baking dough led to the increase of volume compared to the negative control lacking xylanase. At the optimal dosage of 1200 ppm, XYNZG increased the specific volume of 19.1%. The effects of XYNZG on the bread texture profile were also shown in Table 2. With the increase of XYNZG, the initial crumb hardness and chewiness were both improved. The hardness was increased by 17.2% at the dosage of 1200 ppm, while chewiness changed slightly. In addition, XYNZG did not have a significant effect on bread springiness.

**Table 2:** **Effects of xylanase XYNZG on the texture profiles of wheat bread**

**50 U Xylanase dosage (ppm)**	**Hardness**	**Springness**	**Chewiness**
0	334.2±12.0	0.86±0.04	197.6±5.7
800	357.0±2.5	0.86±0.02	206.9±3.5
1200	391.7±3.6	0.87±0.07	211.1±8.0
2000	371.7±15.8	0.86±0.01	215.9±9.3

The measurements were performed after baking with additional 2-hour standby at room temperature. The texture profiles of wheat bread were measured using a Texture Analyzer. Data represent mean±standard deviation of three replicates.

## Discussion

Enzymatic baking serves as a good alternative to improve bread quality and strengthen food safety. Therefore, developing a cost-efficient bio-baking process of bread by enzyme represents a major future trend of baking industry. Many bacteria, fungi, and actinomycetes can produce xylanase while the yield is not very high to apply in industry. The low-cost of xylanase will be a key issue for accelerating bio-baking application in the dough treatment process. So it is important to get the cost efficient and high specific activity xylanase with an improved expression level in an ideal host. To reduce the price of enzymes, finding new xylanase gene and heterologous expression are highly desirable to produce enzymes with better performance. In our previous study, a xylanase gene *xynZG* was cloned from *P. cucumerina* and heterologously expressed in *P. pastoris* with high specific activity of 362 U/ml [12]. Recombinant xylanase XYNZG has optimal pH and temperature of 6 and 40°C, respectively [12]. In spite of the high yield and simple purification process, *P. pastoris* is not permitted for use in the food industry because it is not a GRAS and requires methanol supplement during its fermentation in most condition. Compared to *P. pastoris*, *K. lactis* as a heterologous expression host, which has been used to efficiently express many proteins in the past few years, and has a GRAS status that permits their use in food and feed applications. Thus, in this study, we successfully expressed the xylanase gene *xynZG* in *K. lactis* despite of expression yield in *K. lactis* was lower than that in *P. pastoris.*

So far, several researchers expressed four different xylanase in this system. However, the activity of XYNZG in this study can reach 115 U/ml based on the integration vector, which is much higher than 49 U/ml of XynB from the hyperthermophilic bacterium *Thermotoga maritima* MSB8, which was uniquely expressed stable in *K. lactis* based on the integration vector [11]. The other three xylanase expression in *K. lactis* based on episomal vector would have the risk of instability without selective pressure in spite that their activity are a little lower than XYNZG, 98 U/ml of XynA from the extreme thermophile *Thermotoga* sp. strain FjSS3B [8], 95 U/ml of XynA from *D. thermophilum* Rt46B.1 [9] and 98 U/ml of Xyn11A from *B. halodurans* strain C-125 [10]. Additionally, neither of them was investigated or mentioned about application in baking industry. Their features are not suitable for baking use, either. What’s more, the expression yield of recombinant XYNZG can be further improved through engineered strain containing high copy numbers of the XYNZG, and optimized fermentation condition, making bread-baking application possible.

In addition,xylanase was used to produce xylo-oligosaccharides (XO) in recent years. Owing to the special properties of XOs, they possess a remarkable potential for practical utilization in many fields, including pharmaceuticals, feed formulations and agricultural applications [17]. Among these XOs, xylobiose and xylotriose (DP = 2 and 3) are considered to be the main xylooligosaccharides for food applications since xylobiose and xylotriose can stimulate the growth of intestinal bifidobacteria. The vitro assays showed that both xylobiose and xylotriose can be utilized by *Bifidobacterium spp.* and *B. adolescentis* [13,17]. Contrarily, some harmful microorganisms, such as *Staphylococcus, E. coli* and many *Clostridium* spp*.* cannot utilize xylobiose and xylotriose [18]. Moreover, the sweetness of xylobiose is equivalent to 40% of sucrose while it does not cause increase of blood sugar [17]. In this study, the main products of hydrolytes produced by XYNZG are xylobiose and a small part of xylotriose. The results of baking trial also indicate that addition of XYNZG can increase the bread volume and improve the crumb structure, which is similar to the enzymes in GH11, a previously used industrial xylanase family. Therefore, addition of XYNZG can not only increase the bread volume and the crumb structure, but also improve the flavor and the health value of bread.

## Conclusions

In general, the xylanase gene xynZG can be stably and highly expressed in *K. lactis* as a functional enzyme, and XYNZG can efficiently increase bread volume and improve the flavor and function of bread. In this study, the xylanase gene xynZG of *P. cucumerina* was first successfully expressed in *K. lactis* and the expression activity was the highest activity reported so far. This is also the first report of using *K. lactis* expressed xylanase used in baking industry and the baking experiments showed that addition of this enzyme can efficiently improve the volume and sensory properties of bread.

## Methods

### Strains, plasmids and media

The strain *K. lactis* GG799 (New England Biolabs, USA) was used as the host for the expression of recombinant protein and *E. coli* DH5α was used as the host for cloning and plasmid amplification. Plasmid pKLAC2 (New England Biolabs, USA) was employed as the expression vector of *K. lactis*. LB medium was used for the storage and culture of *E. coli* DH5α, while YPD (1% yeast extract, 2% peptone, 2% glucose) was used for *K. lactis*. Transformants were selected on YCB medium (1.17% yeast carbon base, 0.03 M sodium phosphate buffer, pH 7, New England Biolabs) with 5 mM acetamide and were grown on YPD plates containing 1% RBB-xylan for activity screening. Media YPL (1% yeast extract, 2% peptone, 2% lactose ), YLP (1% yeast extract, 2% lactose, 1.5% peptone), YLPU (1.2% yeast extract, 2.6% lactose,1.2% peptone, 0.3% urea) and YLU (1.2% yeast extract, 2.6% lactose, 0.5% urea) were used for the recombinant xylanase fermentation of *K. lactis* [11].

### Construction of the expression vector pKLAC2-*xynZG*

The *xynZG* (GenBank accession number DQ157736) was cloned previously in our laboratory [12], and the encoding product XYNZG was recorded in UniProt accession Q49UB8. In this study, the *xynZG* was amplified by PCR without the signal peptide sequence, using primers xynF (CCG*CTCGAG*AAAAGAATGGCGCCTGCGACTGATACCCC) and xynR (ATTT*GCGGCCGC*TTAACCAGAGTCCGAAACAGTGATCCTA) with restriction sites *Xho*I and *Not*I (*underlined*), respectively. It was cloned into pKLAC2 vector according to manufacturer’s instructions. The expression vector pKLAC2-*xynZG* was linearized by *Sac*II digestion before transformation, and the transformation of *K. lactis* GG799 was performed by lithium chloride as described by Miklenic et al. [19]. The transformants were screened on the YCB agar plates with 5 mM acetamide, and further selected on YPD plates containing 1% RBB-xylan.

### Expression of the recombinant protein in *K. lactis*

The recombinant strain with the highest halo was named GKX21, and inoculated into a flask containing 50 ml YPD medium, and incubated at 28°C, 200 rpm for 48 h, and then the 50 ml YPD medium was transferred to 50 ml YPL medium for another 3 days. Samples were taken every 24 h for SDS-PAGE and enzyme activity assays.

### SDS -PAGE and zymogram analysis of recombinant XYNZG

The fermentation supernatant was loaded on a 12% polyacrylamide gel for SDS-PAGE, followed by staining with Coomassie Brilliant Blue G-250. The zymogram analysis was performed by the published method of Zhang et al. [20] with birchwood xylan replaced by beechwood xylan (Sigma).

### Determination of xylanase activity

Xylanase were assayed by measuring the reducing groups released from beechwood xylan by the dinitrosalicylic acid method (DNS) [12]. Reaction mixture containing 100 μl of diluted enzyme solution and 2.4 ml of 10 mg/ml suspension of xylan in 0.05 M sodium phosphate buffer (pH 6.0) was incubated at 40°C for 10 min. The reducing sugar was determined by the DNS procedure at the absorbance of 540 nm, using xylose as a standard. One unit of enzyme activity was defined as the amount of enzyme capable of releasing 1 μmol of reducing sugar from xylan per minute under the assay condition.

### The fermentation of xylanase XYNZG in different media

The medium components for XYNZG fermentation were optimized according to the published method with a slight modification [11]. The transformant GKX21 was precultured in 50 ml YPD medium and shaken at 200 rpm and 28°C for 48 h. Then the preculture was transferred into 50 ml media, including YPL, YLP, YLPU and YLU at a concentration of 1%, respectively, for another 96 h culture. The fermentation samples were taken every 24 h to measure the xylanase activities and the biomass.

### Mass spectrometry analysis of xylan hydrolysate

The 5.0% (w/v) beechwood xylan in sodium phosphate buffer (pH 6.0) was hydrolyzed by XYNZG at 40°C for 12 h. The hydrolyzate was centrifuged at 12, 000 rpm for 20 min, and then the supernatant was filtered with 0.22 μm filter membrane (Merck Millipore Ltd). The sample was analyzed with electrospray ion source mass spectrometry in the positive ion reflective mode (Agilent, USA).

### Baking trials

Recombinant XYNZG was tested for its effectiveness in baking applications. The experiment was performed by Applied Technology Center of SUNSON Industrial Group Company Limted, Ningxia, China. The formulation of the bread dough was as follows (per liter): 52 ml of water, 100 g of wheat flour (Ningxia Saibei Company, China), 1.5 g of dry yeast, 20 g of sugar, 1 g of edible salt, 8 g of butter oil, and bread improver (5 ppm Novozymes fungal amylase, 10 ppm DSM glucose oxidase, 10 ppm Novozymes FBG lipase, and 100 ppm Vitamin vitamin C). The various concentrations of XYNZG (50 U, 400 ppm-2000 ppm) were mixed for 6.0 or 6.5 min in a mixer. The dough was then proofed at 38°C for 150 min and baked at 225°C for 20 min.

### Determination of bread characteristics

Bread quality assessment by sensory score is largely based on the personal judgment and the subjective qualitative evaluation. Although the assessment system is not unified, it still reflects the influence of consumer preferences. Sensory properties of control and experimental breads were performed according to the 100-point evaluation system [21] to evaluate different attributes of bread such as visual, textural, biting, and organoleptic characteristics.

In order to avoid the subjective judgment difference, bread quality was also determined on the basis of weight, volume, specific volume, and crumb firmness by instruments. All measurements were performed after baking with additional 2 hours standby at room temperature. The loaf volume was measured using the Volume Measuring Device (BVM-L370LC, Sweden). Crumb texture was measured using the Texture Analyzer (TA.XT-plus Texture Analyzer, USA). Bread was equally sliced into 2 cm pieces, and only three central pieces were used to measure the force required to compress a slice of 1.0 mm/s [15].
